# Estimation of salivary protectin D1 in periodontitis patients with metabolic syndrome following non-surgical periodontal therapy

**DOI:** 10.1007/s00784-025-06514-y

**Published:** 2025-09-08

**Authors:** Teena Selvaraj, Jaideep Mahendra, Nikita Ravi, Pavithra H. Dave, Muskan Bedi, Sivaraj Moti Ram Rao, Pradeep K. Yadalam, Carlos M. Ardila

**Affiliations:** 1 Department of Periodontics, Meenakshi Ammal Dental College and Hospital, Meenakshi Academy of Higher Education and Research, Chennai, Tamil Nadu India; 2 Sri Ramachandra Medical College and Research Institute, Chennai, Tamil Nadu India; 3 Department of Physiology, Government Ariyalur Medical College, Ariyalur, Chennai, Tamil Nadu India; 4 Department of Periodontics, Saveetha Dental College, Saveetha Institute of Medical and Technology Sciences, SIMATS, Saveetha University, Chennai, Tamil Nadu India; 5 Department of Basic Sciences, Faculty of Dentistry, Biomedical Stomatology Research Group, Universidad de Antioquia U de A, Calle 70 No. 52-21, Medellín, Colombia

**Keywords:** Metabolic syndrome, Periodontitis, Non-surgical periodontal therapy, Pro-resolving lipid mediators

## Abstract

**Objectives:**

This study aims to assess periodontal and biochemical parameters and evaluate the salivary Protectin D1 levels in periodontitis patients with and without metabolic syndrome after non-surgical periodontal therapy.

**Materials and methods:**

Forty patients were categorized into two groups: 20 patients in Group P (systemically healthy patients with stage II/III grade B periodontitis) and 20 patients in Group P+MS (patients with stage II/III grade B periodontitis and metabolic syndrome). Parameters including age, gender, height, weight, body mass index, waist circumference, socio-economic status, oral hygiene index (OHI), modified gingival index (MGI), probing pocket depth, clinical attachment levels, fasting blood glucose, HDL-c, total triglycerides, and blood pressure were recorded. Saliva samples were collected before scaling and root planing (PMPR). Full-mouth subgingival instrumentation (SGI) was performed on day 10, followed by reassessment on day 30.

**Result:**

Demographic and baseline periodontal parameters were significantly higher in the P+MS group compared to the P group (p < 0.001). Both groups showed significant improvement in periodontal parameters after PMPR and SGI by the 30th day (p < 0.01). Salivary Protectin D1 levels increased significantly in both groups after treatment (p < 0.01), although no significant difference was observed between the groups at baseline and the 30th day. Protectin D1 levels positively correlated with HDL-c, blood pressure, and MGI at baseline, and with OHI, MGI, PPD, and CAL on the 30th day, but showed no significant association with periodontal parameters.

**Conclusion:**

Periodontitis patients with metabolic syndrome exhibited worse baseline periodontal and biochemical profiles than periodontitis-only patients. Non-surgical periodontal therapy significantly improved periodontal health in both groups, with a concurrent increase in salivary PD1 levels, though no intergroup difference in PD1 expression was observed. While PD1 correlated with HDL-c, blood pressure, and periodontal indices, it did not differentiate the therapeutic response between groups, suggesting PD1 may reflect general resolution of inflammation rather than MS-specific pathways. Further research is needed to elucidate the role of PD1 in periodontitis with comorbid metabolic syndrome.

**Clinical relevance:**

Protectin D1 holds promise as a biomarker for the effective management of periodontitis and metabolic syndrome, potentially aiding in both diagnosis and treatment strategies.

## Introduction

Periodontitis is a persistent inflammatory condition that affects the periodontal tissues surrounding the teeth. Key risk factors for its development include stress, tobacco use, poor oral hygiene, and a variety of systemic disorders. The systemic conditions most frequently linked to periodontitis include metabolic syndrome, diabetes mellitus, and cardiovascular disease [1]. Metabolic syndrome refers to a cluster of biochemical and physiological abnormalities, including increased waist circumference, low plasma levels of HDL-c, elevated plasma triglycerides, high blood pressure, and elevated blood glucose levels [2]. Numerous studies have shown that periodontitis plays a significant role in the onset and progression of metabolic syndrome [3].

The host’s susceptibility to systemic disorders increases with periodontitis, largely due to the accumulation of gram-negative bacteria and the elevated levels of inflammatory mediators such as C-reactive protein (CRP) and interleukin-6 (IL-6) [4]. Therefore, treating periodontal disease not only reduces pro-inflammatory mediators such as IL-6, tumor necrosis factor-α (TNF-α), CRP, and reactive oxygen species (ROS), but also increases the levels of pro-resolving lipid mediators, which are crucial for maintaining homeostasis [5].

Pro-resolving lipid mediators are produced through the enzymatic activation of membrane phospholipids and act by binding to specific G protein-coupled receptors on various cells to regulate immune responses [6]. During the resolution phase of inflammation, these mediators help reduce neutrophil infiltration, lower pro-inflammatory cytokine levels, and increase the recruitment of resolving M2 macrophages. These macrophages clear lesions through efferocytosis without suppressing the immune system. Moreover, pro-resolving lipid mediators enhance the phagocytosis and killing of microbes while possessing both anti-inflammatory and pro-resolution properties [7].

Among the important pro-resolving lipid mediators—such as lipoxins, resolvins, protectins, and maresins—resolvins and lipoxins share similar roles in regulating the resolution of acute inflammation, promoting healing, and acting as receptor agonists that facilitate the resolution of inflammation [8]. Protectins and maresins represent a more recent class of specialized pro-resolving mediators (SPMs), derived from eicosapentaenoic acid (EPA) and docosahexaenoic acid (DHA). These mediators are essential for the active resolution of inflammation. Unlike traditional anti-inflammatory treatments, which block the “on” signal of inflammation, SPMs provide an “off” signal to regulate inflammation without the adverse effects typically associated with anti-inflammatory therapies [9].

Protectin D1, also known as neuroprotectin D1, is part of the DHA metabolome and plays a significant role in altering eicosanoid profiles, thereby influencing inflammation. It has been shown to promote the regeneration of pathologically lost periodontal tissues and to reduce inflammation [10]. However, the role of Protectin D1 in the pathogenesis of inflammatory conditions such as metabolic syndrome and periodontal disease remains underexplored. Limited literature exists on the salivary levels of Protectin D1 in both periodontal disease and metabolic syndrome. Furthermore, the impact of non-surgical periodontal therapy (NSPT) on Protectin D1 expression in periodontitis patients with metabolic syndrome has yet to be fully investigated.

Given the anti-inflammatory and pro-resolving properties of Protectin D1, we hypothesize that periodontitis patients with metabolic syndrome (P + MS) will exhibit lower baseline salivary Protectin D1 levels compared to periodontitis-only patients (P), reflecting impaired resolution of inflammation in metabolic syndrome.

Therefore, the aim of the present study is to determine the expression of Protectin D1 in the saliva of periodontitis patients, both with and without metabolic syndrome, following non-surgical periodontal therapy.

## Materials and methods

### Study design

Sixty patients with periodontitis were initially screened from the Department of Periodontology, Meenakshi Ammal Dental College and Hospital, Chennai, from December 2022 to November 2023, based on inclusion and exclusion criteria. Out of the recruited participants, 20 were excluded (10 refused to participate, and 10 had additional systemic diseases). Finally, 40 participants (both male and female), aged 35–65 years, were selected. The study was explained to all participants in detail, and written informed consent was obtained. The study protocol was approved by the Institutional Ethical Committee of Meenakshi Ammal Dental College (MADC/IEC-1/023/2021).

The participants were divided into two groups:

Group P: 20 periodontitis patients without systemic diseases.

**Fig. 1:**
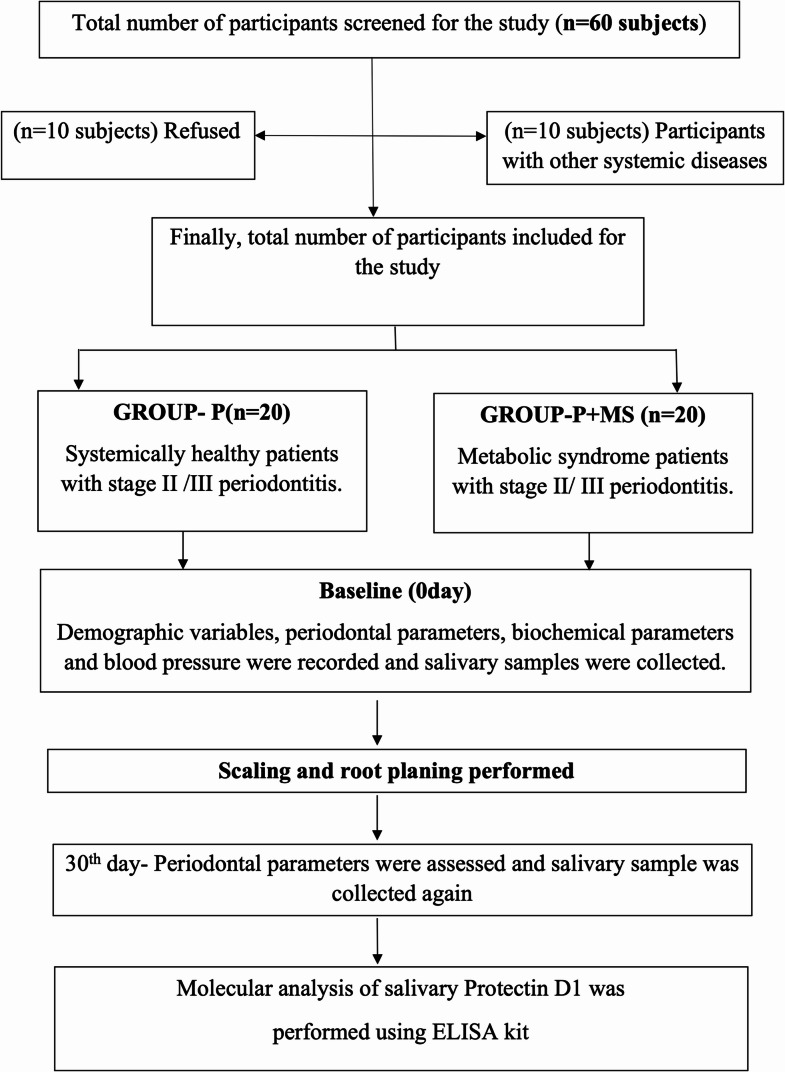
Study design

Group P + MS: 20 periodontitis patients with metabolic syndrome (based on inclusion and exclusion criteria, Fig. 1).

### Inclusion criteria

For both groups, patients were selected based on the 2017 World Workshop classification of periodontal and peri-implant diseases [11]. This included stage II/III, grade B periodontitis with generalized interdental clinical attachment loss (CAL) ≥ 3 mm, radiographic bone loss extending to the middle third of the root and beyond, tooth loss due to periodontitis in ≤ 4 teeth, probing pocket depth (PPD) ≥ 5 mm, and vertical bone loss ≥ 3 mm with Class II/III furcation. Patients with metabolic syndrome were diagnosed using the American Heart Association/National Heart, Lung, and Blood Institute (AHA/NHLBI 2005) criteria: abdominal obesity (waist circumference > 102 cm in men, > 88 cm in women), hypertriglyceridemia (> 150 mg/dL), low HDL-C (men < 40 mg/dL, women < 50 mg/dL), high blood pressure (> 130/85 mmHg), and high fasting glucose (> 110 mg/dL). The presence of three or more of these factors indicated metabolic syndrome [12].

### Exclusion criteria

Patients with a history of antibiotic use, non-steroidal anti-inflammatory drugs, immunosuppressants, bisphosphonates, or systemic steroid therapy in the previous six months, as well as those with salivary gland diseases, systemic diseases (e.g., respiratory, renal, liver diseases, rheumatoid arthritis), allergies, malignancies, HIV or hepatitis infections, pregnant or lactating women, smokers, alcoholics, patients with previous periodontal therapy in the last six months, or patients using nutritional supplements (e.g., docosahexaenoic acid, polyunsaturated fatty acids, monounsaturated fatty acids), were excluded. This study was conducted in accordance with the Declaration of Helsinki (1975, as revised in 2024).

### Sample size estimation

Sample size was determined based on prior research and local hospital records, accounting for the prevalence of metabolic syndrome and periodontitis in the region [13, 14]. Power analysis showed a 95% confidence level with a minimum sample size of 15 subjects per group. To account for potential dropouts, 20 subjects per group were included.

### Demographic and biochemical assessments

A single calibrated investigator (JM) collected dental and medical histories. Another examiner (TS), blinded to the study, assessed demographic parameters including age, gender, height, weight, body mass index (BMI), waist circumference (WC), and socioeconomic status (based on monthly income). Biochemical parameters such as fasting blood glucose, high-density lipoprotein (HDL-C), triglycerides (TG), systolic blood pressure (SBP), and diastolic blood pressure (DBP) were recorded for all participants [15, 16].

### Collection of unstimulated saliva samples

Unstimulated whole saliva samples were collected at baseline and on the 30th day from both groups using the standard spit method prior to periodontal examination. Participants were instructed to avoid food, beverages, and oral hygiene for two hours before sample collection, avoid large meals two hours prior, and abstain from alcohol for 12 h before collection. Samples were collected between 8:00 a.m. and 12:00 p.m. to standardize conditions. Participants rinsed their mouths with water to remove food particles before the collection. Saliva samples were stored at − 80 °C for Protectin D1 analysis [17, 18].

### Assessment of periodontal parameters and non-surgical periodontal therapy (NSPT)

Periodontal parameters (oral hygiene index-OHI- [19], modified gingival index-MGI- [20], probing pocket depth [PPD], and clinical attachment level [CAL]) were assessed at baseline [11]. Full-mouth scaling and root planing (PMPR) were performed, followed by full-mouth subgingival instrumentation (SGI) on the 10th day. PMPR was performed under local anesthesia using ultrasonic scalers and area-specific Gracey curettes. All supra- and subgingival deposits were removed, with particular attention to root surface debridement in sites with PPD ≥ 4 mm. Each quadrant was instrumented with care taken to preserve root structure while achieving complete calculus removal. A second complete SGI was performed to address any remaining deposits and newly exposed calculus (10th day). The procedure again utilized ultrasonic instrumentation followed by manual curettage in all sites with persistent inflammation or residual probing depths ≥ 4 mm. Root surfaces were planed until smoothness was confirmed by explorer examination. All subgingival instrumentation procedures were performed by the same trained periodontist to ensure consistency. Standard oral hygiene instructions (tooth brushing twice daily using the modified Bass technique and interdental cleaning once daily) were provided to all participants. On the 30th day, periodontal parameters were re-evaluated and saliva samples were collected again.

### Molecular analysis of protectin D1 using enzyme-linked immunosorbent assay (ELISA)

Protectin D1 levels were measured using a commercially available enzyme-linked immunosorbent assay (ELISA) [21], following the manufacturer’s instructions, based on a double-antibody sandwich technique. After thawing, saliva samples were analyzed in duplicate. Plates were pre-coated with monoclonal antibodies specific for Protectin D1, incubated with sample diluent, and horseradish peroxidase (HRP)-conjugated antibody for 90 min, followed by development with substrate reagent. Absorbance was measured at 450 nm. The assay standard curve ranged from 0.156 to 10 ng/mL, with a sensitivity of 0.094 ng/mL.

### Statistical analysis

Statistical analysis was performed using the Statistical Package for Social Sciences (SPSS) software version 26.0 (IBM Corp., Armonk, NY, USA). Normality was evaluated using the Shapiro-Wilk test. Inferential statistics were calculated using one-way ANOVA followed by post-hoc tests for pairwise comparisons (Bonferroni correction). The Chi-square test was used to compare proportions between groups. The independent t-test was used to assess differences between baseline and the 30th day after PMPR. Pearson’s correlation was used to assess relationships between variables. Statistical significance was set at *p* < 0.05.

## Results

When comparing the demographic variables between the two groups, age, gender, weight, BMI, waist circumference, and monthly income were significantly higher in the P + MS group compared to the P group (p-value < 0.001) (Table 1). However, no significant difference was found in height and gender between the groups.

**Table 1:** Intergroup comparison of demographic variables among group P and group P + MS at baseline

Variables		Groups			Mean ± standard deviation			*p*-value
Age (years)		Group P			47.60 ± 6.28			0.003*
		Group P + MS			53.85 ± 5.92			
Height (cm)		Group P			163.15 ± 8.02			0.378 ^NS^
		Group P + MS			160.85 ± 8.28			
Weight (kg)		Group P			63.70 ± 6.86			< 0.001**
		Group P + MS			72.45 ± 7.56			
BMI (kg/m^2^)		Group P			24.03 ± 1.46			< 0.001**
		Group P + MS			28.42 ± 2.41			
Waist circumference(cm)		Group P			79.40 ± 5.73			< 0.001**
		Group P + MS			90.40 ± 7.30			
Monthly income (Rupias)		Group P			11600.00 ± 5103.14			< 0.001**
		Group P + MS			20750.00 ± 5077.03			
Gender		Group P		Group P + MS		Total		p-value
		N	%	N	%	N	%	
	Male	9	45.0	11	55.0	20	50.0	0.527^NS^
	Female	11	55.0	9	45.0	20	50.0	
	Total	20	100.0	20	100.0	40	100.0	

*P < 0.05 is statistically significant

**P < 0.001 is highly statistically significant

*NS* Statistically non-significant

Abbreviations: *p* systemically healthy periodontitis, *P + MS* metabolic syndrome with periodontitis

Regarding periodontal parameters at baseline, the oral hygiene index (OHI), modified gingival index (MGI), probing pocket depth (PPD), and clinical attachment level (CAL) were also significantly higher in the P + MS group compared to the P group (p-value < 0.01). Following PMPR and SGI, both groups showed significant improvements from baseline to the 30th day in OHI, MGI, PPD, and CAL (p-value < 0.01) (Table 2).

**Table 2:** Intragroup and intergroup comparison of periodontal parameters among group P and group P + MS at baseline and 30th day following scaling and root planing

Variables	Groups	Baseline	30th day	*p*-value (Intragroup)
		Mean ± standard deviation	Mean ± standard deviation	
Oral hygiene index	Group P	8.72 ± 0.34	1.54 ± 0.12	< 0.01*
	Group P + MS	9.98 ± 0.31	2.943 ± 0.31	< 0.01*
	p-value (intergroup)	< 0.01*	< 0.01*	–
Modified gingival index	Group P	10.54 ± 0.20	3.34 ± 0.32	< 0.01*
	Group P + MS	11.89 ± 0.18	3.96 ± 0.17	< 0.01*
	p-value (intergroup)	< 0.01*	< 0.01*	–
Probing pocket depth (mm)	Group P	6.39 ± 0.28	4.39 ± 0.21	< 0.01*
	Group P + MS	7.85 ± 0.16	4.7 ± 0.28	< 0.01*
	p-value (intergroup)	< 0.01*	< 0.01*	–
Clinical attachment level (mm)	Group P	7.41 ± 0.28	4.73 ± 0.30	–
	Group P + MS	9.40 ± 0.25	6.78 ± 0.22	< 0.01*
	p-value (intergroup)	< 0.01*	< 0.01*	–

*P < 0.05 is statistically significant

At baseline, systolic and diastolic blood pressure, fasting blood glucose levels, high-density lipoprotein cholesterol (HDL-c), and total triglycerides (TG) were significantly higher in the P + MS group compared to the P group (p-value < 0.001) (Table 3).

**Table 3:** Intergroup comparison of biochemical parameters and blood pressure between group P and group P + MS at baseline

Variables	Groups	Mean ± standard deviation	*p* value
Fasting blood glucose level (mg/dL)	Group P	81.50 ± 2.14	< 0.001**
	Group P + MS	87.75 ± 3.86	
High-Density Lipoprotein (mg/dL)	Group P + MS	52.70 ± 4.975	< 0.001**
	Group P + MS	42.50 ± 3.00	
Total Triglycerides (mg/dL)	Group P	160.90 ± 22.75	< 0.001**
	Group P + MS	199.50 ± 5.68	
Systolic Blood Pressure (mm Hg)	Group P	122.0 ± 6.156	< 0.001**
	Group P + MS	128.50 ± 4.617	
Diastolic Blood Pressure (mm Hg)	Group P	79.00 ± 5.52	< 0.001**
	Group P + MS	100.50 ± 8.87	

**P < 0.001 is highly statistically significant

Although no significant difference in salivary Protectin D1 levels was observed between the P and P + MS groups at both baseline and the 30th day, a significant increase in Protectin D1 expression from baseline to the 30th day was noted in both groups following PMPR and SGD (p-value < 0.01) (Table 4).

**Table 4:** Intragroup and intergroup comparison of expression of salivary protectin D1 among groups P and P + MS at baseline and 30th day following scaling and root planing

Variables	Groups	Baseline	30th day	*p*-value (intragroup)
		Mean ± Std. deviation	Mean ± Std. deviation	
Salivary Protectin D1 (ng/ml)	Group P	0.44 ± 0.148	4.98 ± 2.04	< 0.001*
	Group P + MS	0.43 ± 8.50	5.07 ± 2.04	< 0.001*
	*p*-value (intergroup)	0.863	0.888	–

*P < 0.001 is statistically significant

The correlation analysis between Protectin D1 (ng/ml) and various biochemical parameters at baseline revealed weak or minimal positive correlation and no statistically significant associations. Protectin D1 levels showed weak and no significant correlations with oral health parameters at both baseline and the 30th day, suggesting that changes in these clinical markers were not strongly associated with fluctuations in Protectin D1.

## Discussion

Periodontitis is a polymicrobial disease that causes inflammatory changes in the tooth-supporting periodontal tissues, ultimately leading to tooth loss. It affects the host’s susceptibility to systemic disorders by promoting the accumulation of gram-negative bacteria and elevating levels of several inflammatory mediators such as C-reactive protein (CRP) and interleukin-6 (IL-6). Periodontitis has been associated with various systemic conditions, including diabetes mellitus, cardiovascular disease, and metabolic syndrome [22].

Metabolic syndrome is a state of chronic low-grade inflammation resulting from a complex interplay between genetic and environmental factors. It includes insulin resistance, visceral adiposity, atherogenic dyslipidemia, endothelial dysfunction, genetic susceptibility, elevated blood pressure, a hypercoagulable state, and chronic stress. Several studies have evaluated the relationship between periodontitis and metabolic syndrome [23–27]. Treating periodontal disease reduces pro-inflammatory mediator levels such as IL-6, tumor necrosis factor-α (TNF-α), CRP, and reactive oxygen species (ROS) and increases pro-resolving lipid mediators, which are anti-inflammatory in nature and help maintain homeostasis. However, the role of pro-resolving lipid mediators in the association between periodontitis and metabolic syndrome has not been fully explored [24].

Protectin D1 is a lipid mediator that promotes the phagocytosis of apoptotic neutrophils and aids tissue regeneration in periodontal tissue [25]. Although previous studies have explored the levels of Protectin D1 in various inflammatory diseases, its synergistic role in periodontitis and metabolic syndrome remains unclear. To the best of our knowledge, this study is one of the few that evaluates the expression of Protectin D1 in periodontitis patients with metabolic syndrome, using non-surgical periodontal therapy as an intervention. The study aims to assess, compare, and correlate demographic variables, periodontal and biochemical parameters, and the expression of Protectin D1 in periodontitis subjects with and without metabolic syndrome at baseline (0 days) and 30 days after non-surgical periodontal therapy, hypothesizing that salivary Protectin D1 levels increase significantly following PMPR and SGI in metabolic syndrome patients with periodontitis, thereby modulating the inflammatory response and promoting homeostasis.

When comparing demographic variables, the mean age, weight, BMI, waist circumference, and monthly income were significantly higher in the P + MS group than in the P group (Table 1). The mean age was higher in the P + MS group (53.85 ± 5.92 years) compared to the P group (47.60 ± 6.28 years) (Table 1). This finding aligns with Ford et al., who found that most individuals developing metabolic syndrome were aged 50–65 years [26]. Similarly, Kim et al. reported an association between metabolic syndrome and periodontitis in patients aged ≥ 44 years in a Korean population [27]. As age increases, the incidence of local plaque accumulation, calculus, food impaction, and diminished immune function also rises, contributing to an increased incidence of both periodontal inflammation and metabolic syndrome.

In the present study, the mean weight was higher in the P + MS group (72.45 ± 7.56 kg) compared to the P group (63.70 ± 6.86 kg) (Table 1), consistent with Koo HS et al., who suggested that individuals with metabolic syndrome and a weight ≥ 60 kg have a higher risk of periodontitis [28]. Obesity likely increases pro-inflammatory cytokines, insulin resistance, and oxidative stress, all of which contribute to periodontal tissue destruction. Additionally, the mean BMI in this study was higher in the P + MS group (28.42 ± 2.41 kg/m²), agreeing with findings from Saito et al., who reported that a BMI ≥ 25 kg/m² in periodontitis patients increases the risk of metabolic syndrome [29]. Similarly, the mean waist circumference (WC) was higher in the P + MS group (90.40 ± 7.30 cm) compared to the P group (79.40 ± 5.73 cm) (Table 1), consistent with Carneiro et al., who found that increased WC (> 88 cm) correlates with metabolic syndrome in periodontitis patients [30].

Higher monthly income was observed in the P + MS group (Rs. 20750.00 ± 5077.03) compared to the P group (Rs. 11600.00 ± 5103.14) (Table 1). Kim et al. found that rapid economic growth increased the risk of metabolic syndrome in periodontitis patients, possibly due to more sedentary lifestyles associated with higher-income jobs [27]. Height was found to be insignificant between the groups, and gender distribution was equal in both groups, hence not significant.

Among the periodontal parameters, the oral hygiene index (OHI) was significantly higher in the P + MS group (9.98 ± 0.31) compared to the P group (8.72 ± 0.34) (Table 2), in agreement with Mathur et al., who found a higher prevalence of periodontal disease among individuals with poor oral hygiene [31]. The mean modified gingival index (MGI) was also higher in the P + MS group (11.89 ± 0.18) compared to the P group (10.54 ± 0.20) (Table 2), consistent with findings from Nazir MA et al. and Khader YS et al. [32, 33]. Similarly, PPD was greater in the P + MS group (7.85 ± 0.16 mm) compared to the P group (6.39 ± 0.28 mm) (Table 2), as reported by Marchetti E et al. and Nishimura F et al. [34, 35]. CAL was lower in the P + MS group (9.40 ± 0.25 mm) compared to the P group (7.41 ± 0.28 mm) (Table 2), in agreement with Genco RJ et al., who observed greater attachment loss in periodontitis patients with metabolic syndrome [36].

Following PMPR and SGD, both groups showed significant improvements in OHI, MGI, PPD, and CAL at the 30th day compared to baseline, consistent with findings by Torumtay et al., who reported significant periodontal improvements after non-surgical therapy in metabolic syndrome patients [37]. These results suggest that non-surgical periodontal therapy effectively reduces the inflammatory burden and improves periodontal health in patients with periodontitis and metabolic syndrome [38].

Biochemically, fasting blood glucose levels were higher in the P + MS group (87.75 ± 3.86 mg/dL) compared to the P group (81.50 ± 2.14 mg/dL) (Table 3), consistent with Negrato et al., who observed an increased risk of metabolic syndrome in periodontitis patients with elevated glucose levels [39]. HDL-C levels were slightly reduced in the P group (52.70 ± 4.97 mg/dL) but significantly reduced in the P + MS group (42.50 ± 3.00 mg/dL) (Table 3), in line with Kotin et al. [40]. Triglyceride levels (TG) were also higher in the P + MS group (199.50 ± 5.68 mg/dL) than the P group (160.90 ± 22.75 mg/dL) (Table 3), consistent with findings by Tu et al. [41].

Systolic and diastolic blood pressures were significantly higher in the P + MS group (SBP: 128.50 ± 5.52 mm Hg, DBP: 87.75 ± 3.86 mm Hg) compared to the P group (SBP: 122.00 ± 6.15 mm Hg, DBP: 81.50 ± 2.14 mm Hg) (Table 3), aligning with studies by Kawabata et al. and Morita et al., who found a positive association between hypertension and periodontitis [42, 43].

Regarding salivary Protectin D1 levels, a decrease was observed at baseline in both groups, with a significant increase by the 30th day following PMPR and SGD (Table 4). This is consistent with Elabdeen et al., who found lower pro-resolving lipid mediator levels in aggressive periodontitis patients compared to healthy controls [44]. Onal et al. also reported lower salivary Protectin D1 levels in periodontitis patients with cardiovascular diseases, consistent with the present study [14]. However, a recent study observed increased Protectin D1 levels in periodontitis patients, suggesting its anti-proteolytic effect as a protective response to inflammation [45].

At baseline, salivary Protectin D1 positively correlated with HDL-C, systolic BP, diastolic BP, and MGI, indicating its pro-resolving role in reducing inflammation (Table 5). By the 30th day, Protectin D1 again showed positive correlations with OHI, MGI, PPD, and CAL (Table 6), further supporting its protective anti-inflammatory properties in maintaining periodontal tissue integrity and promoting homeostasis [46].

**Table 5:** Correlation of protectin D1 with biochemical parameter at baseline

Variables	Protectin D1 (ng/ml)	
Fasting blood glucose level(mg/dL)	Correlation	−0.116
	P-value	0.475 ^NS^
High-Density Lipoprotein (mg/dL)	Correlation	0.054
	P-value	0.743 ^NS^
Triglycerides (mg/dL)	Correlation	−0.011
	P-value	0.945 ^NS^
Systolic Blood Pressure (mm Hg)	Correlation	0.183
	P-value	0.258 ^NS^
Diastolic Blood Pressure (mm Hg)	Correlation	0.050
	P-value	0.759 ^NS^

*NS* statistically non-significant

**Table 6:** Correlation of protectin D1 with periodontal parameter at baseline and 30th day

Variables		Protectin D1 (ng/ml) Baseline (0 day)	Protectin D1 (ng/ml) 30th day
Oral hygiene index (OHI)	Correlation	−0.146	0.078
	P-value	0.370	0.63^NS^
Modified gingival index (MGI)	Correlation	0.50	0.054
	P-value	0.758 ^NS^	0.742 ^NS^
Probing Pocket depth (mm)	Correlation	−0.008	0.242
	P-value	0.963 ^NS^	0.133 ^NS^
Clinical Attachment Level (mm)	Correlation	−0.128	0.036
	P-value	0.432^NS^	0.828 ^NS^

*NS* statistically non-significant

This study has certain limitations that should be acknowledged. Firstly, the sample size was relatively small, which may limit the generalizability of the findings. A larger sample size would provide more robust data and allow for better statistical power, enabling more definitive conclusions regarding the relationship between Protectin D1 levels, periodontitis, and metabolic syndrome. Excluding smokers and alcohol consumers may limit the generalizability of our findings, as these are prevalent risk factors for periodontitis. However, their exclusion was necessary to reduce confounding effects on periodontal disease progression and systemic inflammation. Additionally, the study only analyzed salivary Protectin D1 levels, whereas serum samples could have offered a more comprehensive understanding of the systemic involvement of Protectin D1 in inflammatory processes. Future studies could incorporate serum analyses alongside saliva to compare the expression of Protectin D1 in both biological fluids, providing a more complete picture of its role in systemic inflammation. Another potential limitation of this study is the inherent heterogeneity within Stage II/III periodontitis, despite efforts to match groups based on clinical parameters. Also the study lacks stratification according to the degree of metabolic control, which may impact systemic inflammation despite all participants being under stable medical management.

Moreover, this study focused solely on a short-term evaluation following non-surgical periodontal therapy. Long-term follow-up studies are needed to assess whether the improvements in periodontal and biochemical parameters, such as Protectin D1 levels, are sustained over time. This would help in understanding the lasting effects of non-surgical periodontal therapy on metabolic and periodontal health, as well as its potential in reducing systemic inflammation.

It is important to note that factors such as age, BMI, and waist circumference—significantly higher in the P + MS group—may independently influence systemic inflammation, potentially confounding the observed effects. Additionally, the bidirectional relationship between periodontitis and metabolic syndrome suggests that metabolic dysfunction could also exacerbate periodontal disease, warranting cautious interpretation of causality.

In terms of future prospects, Protectin D1 holds promise as a potential diagnostic and therapeutic biomarker. Further research should investigate its role in various populations, including different ethnic groups and individuals with other systemic conditions beyond metabolic syndrome. Understanding the specific pathways through which Protectin D1 mediates inflammation could open new avenues for the development of targeted therapies aimed at enhancing its anti-inflammatory and pro-resolving properties. Moreover, its role as a biomarker for monitoring periodontal health could aid in personalized treatment strategies, ensuring timely intervention and improved clinical outcomes.

## Conclusions

This study demonstrated a significant increase in salivary Protectin D1 levels from baseline to the 30th day following non-surgical periodontal therapy in periodontitis patients with metabolic syndrome. This finding highlights Protectin D1’s role as an anti-inflammatory and pro-resolving mediator, suggesting its potential in modulating inflammatory responses associated with both periodontitis and metabolic syndrome. The observed improvement in Protectin D1 levels indicates its utility as a biomarker for assessing the resolution of inflammation and the effectiveness of periodontal therapy. Given these results, Protectin D1 holds promise as both a diagnostic indicator and a therapeutic target. Future research should further explore its potential in early detection and prevention strategies for periodontal disease and metabolic syndrome, with the aim of developing targeted interventions that leverage its anti-inflammatory properties to improve patient outcomes. Periodontitis patients with metabolic syndrome exhibited worse baseline periodontal and biochemical profiles than periodontitis-only patients. Non-surgical periodontal therapy significantly improved periodontal health in both groups, with a concurrent increase in salivary PD1 levels, although no intergroup difference in PD1 expression was observed. While PD1 correlated with HDL-c, blood pressure, and periodontal indices, it did not differentiate the therapeutic response between groups, suggesting PD1 may reflect general resolution of inflammation rather than MS-specific pathways. Further research is needed to elucidate the role of PD1 in periodontitis with comorbid metabolic syndrome.

## Data Availability

Records were obtained from the included investigations.
